# Supplementary material for: Deep causal speech enhancement and recognition using efficient long-short term memory Recurrent Neural Network
Source: PLoS One. 2024 Jan 3;19(1):e0291240. doi: 10.1371/journal.pone.0291240 (PMC10763955; doi:10.1371/journal.pone.0291240)
Supplement: Attachment — Submitted filename: Response to Reviews.docx [file pone.0291240.s001.docx]

The Editor in Chief

PLOS ONE Journal

Subject:	**Response to Reviews**

The authors are thankful that the Editor in Chief, PLOS One Journal and the respected reviewer for their valuable inputs to improve the technical contents of the study. The authors have seriously taken action to answer and address the valuable concerns. The changes of **Reviewer#1** are highlighted with RED colour; for **Reviewer#2**, the comments are highlighted with BLUE colour.

**Reviewer #1: The following revisions are required.**

1. **In the literature review, add 3 to five more relevant and latest techniques.**

**Response**: Thank you for the comment, recent new techniques for SE are included in the revised manuscript. The following literature with references is added to the revised manuscript.

A causal dynamic model using attention LSTM encoder-decoder is proposed for SE with excellent noise reduction and speech recognition results [28]. A time-domain brain-assisted speech enhancement model incorporates electroencephalography signals to extract the target speaker from monaural speech mixtures. The proposed SE model is based on the fully convolutional time-domain network [29]. Another study [30] proposes a cooperative attention-based speech enhancement model and combines local and non-local attention operations in a learnable and self-adaptive manner. The study [31] proposes a multi-scale attention metric generative adversarial network to avoid the mismatch between the objective function used to train the speech enhancement models and introduces the attention mechanism in the metric discriminator. Another study uses a Convolutional attention transformer bottleneck in the encoder-decoder framework for speech enhancement and obtains better SE and automatic speech recognition results [32].

[28]	Peracha, F. K., Khattak, M. I., Salem, N., & Saleem, N. (2023). Causal speech enhancement using dynamical-weighted loss and attention encoder-decoder recurrent neural network. Plos one, 18(5), e0285629.

[29]	Zhang, J., Xu, Q. T., Zhu, Q. S., & Ling, Z. H. (2023). BASEN: Time-Domain Brain-Assisted Speech Enhancement Network with Convolutional Cross Attention in Multi-talker Conditions. arXiv preprint arXiv:2305.09994.

[30]	Xu, X., Tu, W., & Yang, Y. (2023). CASE-Net: Integrating local and non-local attention operations for speech enhancement. Speech Communication.

[31]	Guo, H., Jian, H., Wang, Y., Wang, H., Zhao, X., Zhu, W., & Cheng, Q. (2023). MAMGAN: Multiscale attention metric GAN for monaural speech enhancement in the time domain. Applied Acoustics, 209, 109385.

**2.	Add Comparison table at the end of section 2 and compare with at least 7 to 10 techniques with appropriate parameters.**

**Response**: Thank you for the constructive comment, a comparison Table is added to the revised paper.

**3 Please make sure your paper has the necessary language proofreading.**

**Response**: Thank you for the important comment, the paper is proofread for mistakes.

**4. The conclusion is weak and inconsistent with the evidence and arguments.**

**Response**: Thank you for your constructive comment, the conclusion part is revised according to the arguments in the results and discussions.

**Reviewer #2: Dear Authors, the paper titled “Deep Causal Speech Enhancement and Recognition using Efficient Long-Short Term Memory Recurrent Neural Networks” proposed an hourglass-shaped LSTM capable of capturing long-term temporal correlations via the reduction of feature resolutions without data loss. The paper is addressing important issues however it needs more improvements.

1. Extensive English editing is required throughout the manuscript.**

**Response**: Thank you for the constructive comment, the paper is proofread for mistakes.

**2. Table 2. The details of the network with hyperparameters of the LSTM and competing Deep Learning Models. These hyperparameters require justification on how they have been chosen and how to tune them.**

**Response**: Thank you for the important concern. The proposed model is compared with a well-cited paper and followed the same architectural settings; however, the topology has been changed to get our target results. The learning rate and Adam optimizers with random initialization are used to fine-tune the model parameters. Further, with the same network architecture to the baseline [16], we were certain since that model already provided optimized results in speech enhancement. With our setting, we further improved the results which are confirmed by the results section.

**3. New references need to be updated as very few references are for 2021 and post-2021.**

**Response:** Thank you for the important concern, the references are updated and added several new literatures.

[28]	Peracha, F. K., Khattak, M. I., Salem, N., & Saleem, N. (2023). Causal speech enhancement using dynamical-weighted loss and attention encoder-decoder recurrent neural network. Plos one, 18(5), e0285629.

[29]	Zhang, J., Xu, Q. T., Zhu, Q. S., & Ling, Z. H. (2023). BASEN: Time-Domain Brain-Assisted Speech Enhancement Network with Convolutional Cross Attention in Multi-talker Conditions. arXiv preprint arXiv:2305.09994.

[30]	Xu, X., Tu, W., & Yang, Y. (2023). CASE-Net: Integrating local and non-local attention operations for speech enhancement. Speech Communication.

[31]	Guo, H., Jian, H., Wang, Y., Wang, H., Zhao, X., Zhu, W., & Cheng, Q. (2023). MAMGAN: Multiscale attention metric GAN for monaural speech enhancement in the time domain. Applied Acoustics, 209, 109385.

4. Section 1 Introduction: Deep neural networks (DNNs) are effective ….noise conditions over time. This section should have what makes DNN so famous, and which type of recent applications it has to justify its utilization in the present work. I suggest adding smotednn: novel model for air pollution forecasting and aqi classification; cdlstm: a novel model for climate change forecasting; analysis of environmental factors using ai and ml methods; deep learning based modeling of groundwater storage change; deep learning-based supervised image classification using uav images for forest areas classification.

**Response**: Thank you for the important suggestion, the authors have added other applications of deep learning into the revised manuscript.

5. Rewrite section 3.4 Representation of Algorithm.

**Response**: Thank you for suggestion, the section 3.4 is revised.

Various SE systems are designed with an interpretation indicating the neural network type, with and without skip connections and mask type. (i): LSTM-NoSkips-IRM: This model estimates the IRM training objective by using the proposed LSTM without skip connections. (ii): LSTM-WithSkips-IRM: The model estimates the IRM by using the proposed LSTM with skip connections. (iii): LSTM-AttenSkips-IRM: This model estimates the IRM by using the proposed LSTM with attention skip connections. The baseline LSTM [16] is represented as LSTM-IRM with IRM as a training target. TIMIT, LibriSpeech, and VoiceBank datasets are used to train all networks

**6. # of parameters is required for the models with FLOPS and the computational complexity.**

**Response**: Thank you for the suggestion, the complexity and parameter count is added to the revised manuscript.

The computational load of the proposed model is measured with trainable parameters and FLOPs (floating-point operations), useful metrics for calculating computational complexity and optimizing the performance on specific hardware platforms. The parameters count and FLOPs for the proposed LSTM model are 26.47M and 127.72 [G] whereas the parameters count and FLOPs for the baseline LSTM are 53.93M and 245.67 [G], respectively indicating the better performance in terms of model complexity and trainable parameters.

**7. Limitations and the future scope should be added with more clarity.**

**Response**: The limitations and future scope are revised. Thank you.

Phase plays a vital role in improving perceptual speech quality whereas a complex Phase spectrum can add significant quality and intelligibility improvements in speech enhancement systems [49]. The focus of this study is to estimate speech magnitude enhancement where the noisy phase is used during speech waveform reconstruction. Our future study will focus on the simultaneous estimation of the magnitude and phase of the speech and intend to integrate the estimates with the proposed model topology. Further, robust loss functions and feature sets will be developed for improved speech quality and intelligibility
